# 

**Published:** 1871-09

**Authors:** 


**ORIGINAL CONTRIBUTIONS.**

Homœopathy as it Was and Is. A Paper Read before the Chicago Medical Society, July 17, 1871. By Charles W. Earle, M.D. 517

Case of Protracted and Complicated Disease. By L. D. Robinson, M.D., Brookfield, Mo. 546

Diseased Kindneys in Scarlatina—Experimental Studies thereon. By Dr. Carl Proegler, Aurora, Ill. 551

Syphilitic Paralysis. By Lyman Ware, M.D., Chicago, Ill. 554

Treatment of Lupus Exedens. By E. Andrews, M.D., Prof. of Surgery in Chicago Medical College. 559

**BOOK NOTICES.**

On Some Diseases of the Nervous System in Childhood. By Charles West, M.D., etc. 570

Braithwaite's Retrospect of Practical Medicine and Surgery 570

American Journal of Obstetrics and Diseases of Women and Children 570

**CORRESPONDENCE.**

Rare Cases. By Alfred A. Castleman, M.D. 560

**PROCEEDINGS OF SOCIETIES.**

Æsculapian Society of the Wabash Valley 562

**EDITORIAL.**

Public Medical Library 570

Cundurango 571

Circular. Illinois Institution for the Education of Feeble-Minded Children 571

The Cholera 572

Cholera at Antwerp 573

The Medical Register and Directory of the United States 573

One of the Horrors of War 573

Calabar Bean in Tetanus 574

Mortality for the Month of July, 1871 575

Money Receipts 576

Belladonna Plaster 576

